# Working through multiple crises: the experience of psychotherapists and psychoanalysts in Lebanon

**DOI:** 10.1186/s40359-024-01810-w

**Published:** 2024-05-28

**Authors:** Rose Marie Nassif, Mayssa’ El Husseini, Nour Beaini, Tonnie Choueiri, Layla Tarazi-Sahab, Marie-Rose Moro

**Affiliations:** 1 University Sorbonne Paris Nord, Villetaneuse, 93430 France; 2 UVSQ, Inserm, CESP, Team DevPsy, University of Paris-Saclay, Villejuif, 94807 France; 3 University of Picardie Jules Verne CHSSC EA 4289, Amiens, France; 4 Saint Joseph University, Beirut, Lebanon; 5 APHP, Hôpital Cochin, Maison de Solenn, Paris, 75014 France; 6 University of Paris Cite, PCPP, Boulogne-Billancourt, 92100 France

**Keywords:** Covid 19, Setting, Trauma, Crisis, Counter-transference, Meta-frame

## Abstract

**Introduction:**

This research explores the impact of the COVID-19 pandemic on psychotherapists' practices and their ability to maintain a framework despite a shared reality with their patients. The specific focus in this article is on the Lebanese context, which is characterized by a series of crises including economic collapse, the COVID-19 pandemic, and the Beirut blast. The objective of this study was to examine how the destabilization of the meta-frame due to crises necessitates adaptations in theoretical knowledge, practice, and setting.

**Methods:**

We conducted a qualitative study among a population consisting of mental health professionals, which were recruited in Lebanon through associations and societies of psychologists, psychotherapists, and psychoanalysts. Data was collected using semi-structured individual interviews. The interviews were analyzed using interpretative phenomenological analysis (IPA), which allowed for a dynamic exploration of the participants' experiences.

**Results:**

Our study revealed four superordinate themes: (1) The strained frontiers; (2) The cumulative traumatic reality and its impact; (3) A challenged professional identity; (4) The creativity stemming from collective trauma.

**Conclusions:**

Our results highlight the insecurity caused by external reality infiltrating the therapeutic setting. Online therapy allowed for continued work, but uncertainty about the online environment's impact on therapeutic relationships was observed. The study underscores the importance of adaptability, containment, and support for therapists navigating crises, particularly in the online setting.

In the face of crisis, mental health professionals in Lebanon navigate unprecedented challenges. The COVID-19 pandemic, economic collapse, and Beirut blast have reshaped the therapeutic landscape. This study examines how psychotherapists in Lebanon adapt to these crises, exploring themes of professional identity, therapeutic boundaries, and creative responses. Through qualitative analysis, we provide insights into navigating uncertainty in mental health practice amidst multifaceted adversity.

## Introduction

A series of nationwide protests erupted on 17 October 2019, in response to the government's failure to find a solution to the economic crisis that has threatened Lebanon for nearly a year. The protests came directly after proposed strategies by the government to increase state revenue for 2020: the announcement of the implementation of new taxes on petrol, tobacco and online calls through apps like WhatsApp [1]. Since this date Lebanon’s economic, social and security situation has rapidly deteriorated and is now facing a “triple crisis” caused by the economic meltdown, the global COVID-19 pandemic and Beirut blast on August 4th, which destroyed its capital.

The World Bank published in 2021 that Lebanon is experiencing an "economic and financial crisis [that] could be ranked among the 10 or even the 3 most severe global crises since the mid-nineteenth century" [2]. This is accompanied by shortages of petrol, medicine, food and health products.

“As a result, a wide segment of the Lebanese and non-Lebanese population has fallen below the poverty line, with livelihoods of those already living in poverty worsened due to inflation, a decline in employment opportunities, and a reduction in basic social service provision.” [3].

Moreover, Lebanon is on the one hand located in an unstable geopolitical area; on the other hand, it is a country that is itself experiencing political and security instability. In this regard, contemporary Lebanon has experienced a series of wars: a civil war between 1975–1990, the Lebanese-Israeli war of 1996, the liberation of the South in 2000 and the Lebanese-Israeli war of 2006. Also, several attacks and assassinations during the years 2005–2006 shook the population [4]; in 2015, ISIS car bombs shook Beirut and killed dozens. It is in this context of multiple crises, and with this history, that the participants work in Lebanon.

Puget and Wender [5] propose the term "superimposed worlds" to characterize situations in which there is a risk of distortion and transformation in listening and in the analytical function when traumatic facts are found in the session material [6]. In countries where we note the prior presence of conflicts, the convergence between global health issues and political situations would exacerbate existing crises and leave the population in the grip of its own anxieties, without a regulating third party. The political and societal reality would impact psychotherapists, and in particular their way of dealing with their framework to continue working with their patients.

The issue of the asymmetry between the patient and the psychotherapist, both of whom are immersed in a world outside of fantasy' [7] arises, highlighting a weakening of the framework and boundaries between patient and therapist. In effect, this asymmetry is at the foundation of the working alliance. Boulanger [8] cited by Nuttman- Shwartz and Shaul [9], proposes the fact that when the personal and professional cannot be separated after shared trauma, it challenges this necessary asymmetry.

Kaës first introduced the meta dimension of the frame in 2012 [10]. He revisits various authors and psychoanalysts including: Bion, Pichon-Rivière, J. Bleger, Anzieu and Gibelot [10] and formulates the principles of container and meta-container associated with the meta-frame. The container is essentially a function. It allows for the containment and transformation of elements of the psyche. A meta-container is the context that enables and supports the existence of a container [10]. Pandemic, war, natural disaster, revolution, socio-political crisis, economic crisis and many others, disrupt the external reality and impose changes and destabilize the meta-frame. The psychotherapist needs to find ways of adapting both his theoretical knowledge and his practice and setting.

When the meta-frame is destabilized, psychotherapists resort to the relationship with their patients to continue working. Indeed "what is engaged in them is also their internal world as well as the intermediate world, that of an in-between space that is created in the therapeutic relationship" [11]. This space contains the objects and feelings remobilized in the therapeutic process. Being preserved despite the break-in of the meta-frame, this link ensures the function of ‘containing shell and excitatory shield’ as defined by Anzieu [12]. A function essential to the maintenance and continuation of therapy.

When the outbreak of COVID-19 was proclaimed a pandemic in March 2020 [13], psychotherapists around the world had to comply with the restrictions imposed, which resulted in either treatment interruptions or a shift to remote therapy. Although psychotherapy, like other medical treatments, was one of the few exemptions to the full curfew, remote treatments did increase. There has been a research interest in remote psychotherapy before the pandemic, the topic has now become a “major contemporary issue” [14]. Simpson and Reid [15] conducted a literature review on research studies that measured therapeutic alliance over 23 years. Their findings support the notion that therapeutic alliance can be developed in psychotherapy by videoconference at least as highly as in a face-to-face setting. Norwood and al [16] in their systematic literature review and meta-analysis also demonstrated good working alliance and outcome for Videoconferencing psychotherapy. Bekes and Aafjes-van Doorn’s [17] findings suggest that psychotherapists’ thoughts about online psychotherapy are related to their past experiences, such as clinical experience, therapeutic approach and varies if they have previous online psychotherapy experience as well. Mitchell's [14] literature review of research in the field of online therapy and counseling highlights the absence of qualitative research aimed at seizing the subjective experience and investigating the profound meanings of the online experience of a psychotherapist.

Our aim is to understand how an external shared reality can modify the psychotherapist's working methods, his listening capacity, his observations with his patients. In other words, we explore the impact of the invasion of the “meta-frame” [10] in the clinical setting.

In this paper we focus on the Lebanese reality and its specificity. The heightened level of stress caused by the concurrence of multiple crises makes Lebanon a particular context that allows us to put into perspective the impact of the Covid 19 crisis on the practices of psychotherapists in other countries.

## Materials and methods

This study is part of an international research project involving researchers from Lebanon, France, Italy and Brazil.

This research project was born, during the Covid 19 health crisis, from the experience of psychotherapists and psychoanalysts who were forced to question and adapt their practice and their setting in order to continue their work with their patients (distance from the patient, confinement, online sessions, etc.).

This pandemic raised many questions about the sharing of reality between a psychotherapist and his or her patient.

This is an exploratory qualitative study approved by the research ethics board of CER U-Paris.

N° IRB: 00012020–65 and the research ethics board of the Saint Joseph University in Beirut USJ-2020–214.

### Methodology

In this study, we used Interpretative Phenomenological Analysis (IPA) [18], a qualitative method based on the principles of hermeneutics and phenomenology. IPA operates within a 'double hermeneutic' in which researchers interpret participants' interpretations of their own experiences, recognizing the subjective nature of human understanding and the dynamic interplay between researcher and participant perspectives. Based on phenomenological research principles, IPA focuses on the in-depth exploration of the lived experiences of individuals in order to capture the essence of their subjective reality. Central to IPA is its idiographic nature, which emphasises the unique and individual nature of participants' experiences rather than seeking to generalise findings across populations. Through a systematic and iterative process of data collection and analysis, IPA enables researchers to uncover the rich and nuanced meanings that individuals attach to their experiences, shedding light on the complexities of human perception and interpretation within specific contexts.

### Participants

The participants were recruited through a purposeful sampling [19], widely used in qualitative research. The selection of the participants was conducted according to the following inclusion criteria:

- Being a Lebanese mental health professional
- Practicing in Lebanon
- Having maintained on going treatment or follow-up with adult and/or child patients throughout the confinement and crisis period in Lebanon.

The recruitment of participants took place in Lebanon through associations and societies of psychologists, psychotherapists and psychoanalysts.

In all 15 participants agreed to take part in the study and were interviewed. Participants’ characteristics are described in Table 1.

**Table 1:** Characteristics of participants

Participant	Sex	Age	Country of origin	Country of practice	Profession/method	Population
P1	F	64	Lebanon	Lebanon	Clinical psychologist/psychoanalysis	Adults/teens/Children
P2	M	44	Lebanon	Lebanon/France	Clinical psychologist/CBT	Adults/couples
P3	F	34	Lebanon	Lebanon	Clinical psychologist/psychoanalysis	Adults
P4	M	44	Lebanon	Lebanon	Clinical psychologist/EMDR/NLP	Adults/teens/Children
P5	F	43	Lebanon	Lebanon	Clinical psychologist/Psychodynamic therapy	Adults/teens/Children
P6	F	44	Lebanon	Lebanon	Clinical psychologist/psychoanalysis	Adults
P7	F	49	Lebanon	Lebanon	Clinical psychologist/psychoanalysis	Adults/teens/Children
P8	F	38	Lebanon	Lebanon	Clinical psychologist/Psychodynamic therapy	Adults/teens
P9	F	42	Lebanon	Lebanon	Clinical psychologist/psychoanalysis	Adults/teens/Children
P10	M	63	Lebanon	Lebanon	Clinical psychologist/psychoanalysis	Adults
P11	F	34	Lebanon	Lebanon	Clinical psychologist/Psycho organic therapy	Adults
P12	F	35	Lebanon	Lebanon	Clinical psychologist/psychoanalysis	Adults
P13	F	40	Lebanon	Lebanon	Clinical psychologist/Analytic therapy	Adults
P14	F	34	Lebanon	Lebanon	Clinical psychologist/CBT	Adults/teens/Children
P15	M	43	Lebanon	Lebanon	Clinical psychologist/psychoanalysis	Adults

*CBT* Cognitive behavioral therapy, *EMDR* Eye Mouvement Desensibilisation and Reprocessing, *NLP* Neuro-linguistics programming

### Measures

The participants were notified about this research by an information letter and a meeting was set up, during which they received clear oral and written information and provided written consent. The data were collected in semi-structured interviews, each about an hour, conducted by the research team. The research team developed the interview guides during meetings and adapted them progressively during the study. Table 2 describes in details the thematic areas explored in the interview.

**Table 2:** Interview guide

**I - Impact of the external situation (Covid/confinement) on the participants **
1- Could you tell me how you experienced the news of the pandemic and the lockdown?
2- With what images would you describe the lockdown?
3- What did you observe in yourself as emotions, behaviors, physical changes?
4- What impact did this have on your creative abilities?
5- Did this situation bring back memories of similar experiences?
**II- Impact of the situation on participants’ work with clients **
6- What decisions did you take to ensure continuity of work?
7- How did you put in place virtual/distance work?
8- What impact did online work have on you? On your patients?
9- What were your habits concerning clients? How did these habits change with online work?
11- How did your ability to adapt and to transform things creatively come into play during the lockdown?
12- Did you notice changes in your way of being? of doing? (Posture, ways of interacting, communicating, relational strategies)
13- How was the notion of intimacy affected?
14- Was there an invasion by patients into the private space? Did you observe their environment more intensely?
15- What difficulties do you meet with online work with patients?
16- With the changes to your life rhythm, was the time allotted to sessions respected? (Slowness, change of time, technical and material issues)
17- How did the notion of debt come into play? (Think of link with payment modalities)
18- What impact did the absence of the body of your patient in the workspace have on you? What impact on the patient?
19- What are the challenges of working in this situation? What are the advantages of working online or remotely?
20- How did you maintain the connection and interaction with your colleagues?
**III- Impact of the situation on countertransference**
21- Could you tell me about your dreams? The patients’ dreams?
22- What images come to you while listening to your patients?
23- Could you describe more specifically the unfolding of a course of therapy that marked you?
24- Could you tell me what you experienced (feelings, thoughts, physical reactions) when a patient would tackle the subject of confinement, of the disease? (Distinguish if this is linked to previous context of political instability, economic crisis etc.)
25- In what ways could you relate to some of the patients’ experience?
26- How did you feel about your internal availability and your ability to listen?
27- What are your thoughts about potential traumatic resurgences or trauma traces in patients after the pandemic and the confinement?
28- What do you think about traumatic resurgences or potential trauma traces in yourself after the pandemic and the confinement?
29- Do you think that new elements will emerge in your relationship with your patients after this situation?
**IV- After the confinement?**
30- How do you imagine the end of the confinement? (What pictures come to mind, ideas, thoughts)
31- How do you imagine going back to work after the confinement? What difficulties could you encounter with your patients?
32- Would you be more open to accept that patients do their therapies mostly or exclusively online?
**V- Impact of Beirut explosion **
33- Could you tell us about your situation at the clinic at the moment of the explosion on the 4^th^ of August? *(Clinical situation) *
34- Could you describe the damages and possible injuries that resulted after the explosion?
35- When and how did you go back to clinical practice after this event?
36- How would you describe your feelings, your thoughts and your experience during the period that followed the explosion of the 4^th^ of August?

### Data analysis

The analysis followed a structured approach using the IPA methodology [18]. Initially, all interviews were meticulously transcribed and subjected to individual scrutiny. Throughout the analysis process, the authors periodically cross-referenced various stages to ensure consistency. Adopting the iterative nature of IPA analysis, emphasis was placed on fostering a dynamic interaction between the researcher and the textual data. Following transcription, repeated readings of the text were undertaken to immerse oneself in the participants' narratives. This involved paraphrasing each interview and annotating interpretations, insights, and interconnections within the text. Subsequently, subordinate themes naturally surfaced, indicative of the depth within specific passages. These subordinate themes were then clustered based on content, giving rise to overarching superordinate themes that encapsulated the collective meanings of the subordinate themes.

To ensure the validity of our research, the interviews were analyzed independently by three researchers (RN, TC, and NB) and the divergences discussed in research team meetings.

## Results

The analysis of the data revealed four superordinate themes: (1) The strained frontiers; (2) The cumulative traumatic reality and its impact; (3) A challenged professional identity; (4) The creativity stemming from collective trauma. Each superordinate theme came with constituent subordinate themes. These are described further within this section.

### Strained frontiers

#### Intrusion of the external threat into the setting

The outside world is perceived by the participants as dangerous. *"Everything outside was a threat and there was the question of when we could go outside and when you had to go inside." P8.* Whether it’s the Covid 19 virus, the socio-political situation, the explosion; the external reality could at any moment break into the setting. *"The danger was there, it was there, more outside than inside". P1.*

“*There are not only attacks from within, this year we had attacks from outside, which you can neither decide nor control, whether it's the virus, or socio-political and economic worries, and then the explosion.” P3* It is as if the frontiers between inside and outside were becoming porous.

#### Collapse between the family and the professional sphere

For some psychotherapists, having to work online meant having to work from home, making it difficult to separate the family sphere from the professional sphere.

*"I was also stressed because at the family level I had to take care of a lot of things in my head, so I had to go to the supermarket, I had to wash things, I had to make sure there were vegetables and fruits, so it was a whole logistical thing for me, along with doing the sessions from home" P5.*

*“We stopped doing field work, we did the sessions by phone. And that was very difficult because I had two children at home and we had to do the sessions during the day, so it was very difficult to keep the children plus do the sessions.” P11.*

*“I had found it difficult to manage with a child who had just been born at the time. Then they said it was two weeks and I thought how are we going to spend two weeks all together at home when I had to keep working?” P15.*

#### Resonance between the psychologist's experience and that of his or her patients

For some participants, experiencing a common shared reality with their patients, made them question the impact of this resonance on the setting.

*" The patient knows that I had to stop the face-to-face sessions because I am subjected, like him, to the same external rules of the Ministry of Health, like him, I am subjected to the inconsistencies sometimes of my government, revolution, economic crisis, since we have also stopped payments in dollars, all these inconsistencies, we live them, at the same time and they necessarily enter into the setting of the therapy " P7.*

They identify feeling distraught to share the same reality with their patients, erasing the asymmetry specific to the therapeutic relationship. *"The patient is affected and I am also affected, it's not like before, we are equal and I find it catastrophic. “P6.*

### The cumulative traumatic reality and its impact

#### The accumulation of crises

In the case of countries such as Lebanon, the health crisis came on top of an already ongoing social, economic and political collapse. Worrying about covid was not a priority for most participants who reported being affected by the cumulative effect of the multiple crisis occurring.

*“My worries were or still are related to the country's crisis. Or the crisis that we are passing through, starting from the electricity, the inflation, the explosion, and uncertainty. “P4.*

*“For everyone it was one thing, which was the pandemic, and for us it was several things: the road cuts, then we had the petrol and fuel shortage, then we had the electricity shortage, then we had everything. And then inflation too.” P15.*

*" I think it's a very, very special year in Lebanon, as it is for everyone, and then after the explosion, we had to work with a new form of anxiety, which comes on top of the virus, which is that in a few seconds you can lose everything, and especially your life. So, we had to work with another wave.” P3.*

#### Resurgence of war images and traumatic experiences

Most of the participants identify the resurgence of war related images when asked about their experience of the confinement.

*"The atmosphere in the supermarket that day, it's been too long but I remember, people in masks, people who are afraid of each other, who move quickly, who avoid eye contact, well, looking into each other's eyes, it was an atmosphere of war" P5.*

*"I found myself after this video call, with my memories of when I was in the, when we were at school and there were bombings, when I was in the shelters.” P2*

Moreover, the resurgence of the mentioned traumatic experiences is extended to the wider reality enclosing the uncertainty of the economic collapse and that of the explosion of the Beirut port.

*"I have war reflexes. When I was a child, we lived in Beirut, and we had a house in the mountains, and every time there were explosions like that, we kept all the windows open, it was a reaction. So that's what I did in my house here. Therefore, we didn't have any damage, although the whole building was blown away." P6.*

*“Related to the explosion for example, seeing people waiting turns for gasoline, also for bread, a few months ago, so all these images like they bring back the civil war stories and experiences that we had.” P4.*

### A challenged professional identity

Having to work in a potentially traumatic context of multiple crises and struggling with the resurgence of past traumatic experiences, participants identify their professional identity being challenged as a central element of their experience.

#### Availability and listening capacities shortened or bypassed

The participants mention a submerged capacity to listen, to be available and to contain the patient.

*"What bothered me was that I couldn't hear the phantasmatic content and what was coming from the unconscious because I was so afraid.” P9*

*"I took a long month for myself to be able to be more ready, to be able to listen and not be overwhelmed, because I was completely paralyzed in my head and overwhelmed, completely. The framework, the space that ensured a certain continuity was no longer there at least with me, I had to make do with what was still left, what still existed." P3.*

They question themselves about the quality of their work, as the line between resistance to the treatment and the reality are blurred in these circumstances (financial difficulties making it impossible to pursue the therapy).

*"For example, a patient who says yes I might not be able to come anymore because I don't have any money, and for me I just interpreted that in my head as resistance, and when she said she wanted to stop, I thought ok let her stop. It's like I really feel I've missed out." P.13.*

*“As a psychotherapist we question the quality of the work we are doing with these people when we feel relieved when a session is canceled.” P.14*

#### Massive insecurities related to the therapist’s feeling of unreadiness and inefficiency

Participants report feeling insecure in relation to changes in their therapeutic method or approach.

*"It felt like you were starting to want to guide people versus do therapy. And it was tricky as well to make an effort not to do that." P8*

*"After the August 4th explosion, I felt I had an urgency to interpret in the sessions, as if I was in a hurry. I found myself thinking I want to solve all his problems now." P9*

It felt more challenging for younger therapists to maintain and reinforce their therapeutic framework.

*“I sometimes questioned my work, and because the sessions were skipped so easily at times, it was even easier for me to postpone sessions to another day or another time because the framework was very shaky. It was very difficult for me to adapt to that. And honestly, in the first few weeks, the framework was very difficult to set up for the online sessions.” P14.*

*“I was in psychoanalytical training, and it was the beginning, so I had to be the guarantor of the framework and the posture and of the person. It was very disturbing because it was new, I had to internalize it and master it, in order to move on to something virtual. I was like a baby taking its first steps, but I felt that everyone around me was also taking its first steps, those who weren't used to doing sessions online.” P12.*

Some participants also mention a certain frustration in regards to the payment of sessions when manipulation and bargaining become possible following the inflation and shifting to online work.

*"There are possible manipulations, when you are at a distance. It's like they take you for granted. Kind of like what did you do, what did you bring to us?" P9*

*"For this patient it was the telephone sessions that he paid half price and the face-to-face session, he paid it as before, I said ah but why?” P1*

### Creativity stemming from collective trauma

#### Measures taken to resume work with patients

Our participants who are attacked by the external reality in their professional identity, their setting and their practice seemed to have found ways to avoid suffering from impotence and helplessness in the face of this castrating reality.

*“I felt I was more Winnicottian during the pandemic, and during all what's going on in Lebanon as well, which can sometimes be worse than the pandemic. But I didn't lose sight of the analytical aspect of things. The analytical aspect in the sense of the analytical technique." P10.*

*"The explosion was on August 4. I went back to work on August 7, without windows or curtains, but I wanted to go back to work anyway, to show the patients and myself above all, that it is still there, and that it is continuing.” P3.*

*“We were confronted with challenges which were really sources of creativity that we had not suspected in ourselves and it is all very well to say that psychoanalysis and psychoanalysts and all those who are interested in it are a bit rigid, a bit overwhelmed by time, by events *etc*. by the course of modern life *etc*., but we found that it was not the case.” P15.*

#### The shift to online work was an adapted strategy to counter the economic crisis

The participants showed great creativity in arranging their settings to ensure continuity in the follow-up of their patients and even to take on new patients. Most participants expressed relief at being able to continue working, and for some the continuity was even described as life-saving.

*"In a way it was a tool that was introduced by the pandemic but which was in the service of trying to compensate a little for the economic crisis and having access to patients who live abroad and who pay in foreign currency, so it was in the service of that.” P.12.*

They also resorted to self-care by finding physical activities or creative activities or by implementing a support system with peers in order to prioritize their wellbeing so that they could be « available» for the patients.

*" I'm lucky enough to have participated in a writing group very early on, and so that was my only creative passion as a psychoanalyst because we were talking about practice as an analyst in confinement, so I started to write in this group and to… to read and write, around this subject and that's it" P7.*

*That's what we did, that's it, live shows, seminars, conferences, but sometimes even on a personal level, with two or three female colleagues, so we were in contact all the time, almost every day" P3.*

*"I had headaches, much more than usual, that's why I was doing yoga, yoga stabilized me physically." P6*

#### Supervision and resuming analysis

Some participants took up personal analysis / therapy sessions or even supervisions. For the Lebanese psychotherapists it was important to be able to work with foreign therapists, the triangulating foreigner.

*"Last winter, and until now, I needed this alternative space for reflection and so I resorted to a psychoanalysis in Turkey, and so, I felt that I needed this space or this external container outside of Lebanon to pick myself up." P12.*

*“We had group therapy after Beirut explosion, EMDR group therapy done by our trainer who is from UK. It was online group therapy. If it wasn’t for that, I think I wouldn't have been able to do trauma therapy for those who were traumatized from the Beirut explosion”. P4.*

*"I sometimes felt that the listening ability of the Lebanese supervisors was operative. And so, I felt that we were all in this same mode of operation and I was only able to extricate myself with a foreign supervisor who doesn't live in Lebanon." P13.*

## Discussion

The aim of this study was to explore psychotherapists’ lived experiences of working through multiple crises. The qualitative analysis of the therapists’ interviews revealed that the core aspect of their experiences is the feeling of insecurity related to the effraction of the external reality into their setting and framework.

The switch to online therapy, although a forced transition and for most participants a first experience, allowed them to continue working with their patients and even take on new patients. They expressed relief and felt that this was an appropriate strategy for dealing with the economic crisis. However, our results highlight the participants' uncertainty about the online setting and its impact on the therapeutic relationship with patients. In the literature in the psychodynamic and psychoanalytic oriented research community, remote psychotherapy has been discussed on the basis of the theoretical concepts and approaches that form the basis of psychodynamic practice, such as transference, countertransference, resistance, emotional attachment, containment, etc. On this, Jesser et al. [20] refers here to Sharff [21], Bayles [22], Lemma [23]. Sharff [21] reviews the psychoanalytic literature and shows the development of analytic thought on the practice of technology-assisted psychoanalysis to back up her case that analysis using the telephone and the Internet is a valid and a clinically effective alternative to traditional analysis when needed. Bayles [22] argues that as psychoanalysis increasingly relies on technology, we must consider the implications of how the limitations of information communicated by the body in the nonverbal realm influence the analytic encounter. For Lemma [23] generalities have limited value. She suggests that we need a psychoanalytic lens to focus on the individual's psychic capacity to cope with what is presented or staged in a given virtual space.

For our participants, having to do their online sessions from home resulted in the collusion between the professional sphere and the family sphere. Jesser et al. [20] found that “working from home and mixing personal and professional spheres meant that there was little time to tune into or reflect on sessions.”

Therapists also mentioned slight modifications and adaptations of their working methods or therapeutic approach. This has been pointed out by Velykodna and Tsyhanenko [24]. Changes in psychoanalytic stance were noticed, “as practitioners became more supportive, careful, cautious, manageable, through feeling something close to “primary maternal preoccupation” (in Winnicott’s terms, 1957), wherein they were ready to give advice and reduce the regular fee.” [24].

Under the specific circumstances of our participants, the framework is being challenged in terms of frequency, fees, content, and resistances. “When the economic crisis intrudes on the analytic dyad, complex transference–countertransference ‘dynamics’ (vs. “vicissitudes”) are set forth that require great care in order for the analytic relationship to survive without deformation.” [25].

Kogan, quoted by Christopoulos [25] discusses the idea of a defensive denial of external reality by the psychoanalyst in an effort to neutralize his or her feelings of distress and helplessness in the face of the traumatic effect of external reality. It is essential, she says, to recognize this reality in order to be able to ensure a containing function and consequently to be able to explore the patient's internal response to this external situation and its entanglement with his personal history and functioning.

In agreement with A. Green [26] who proposes that when the setting needs to be drastically changed, the therapist should turn to an internal setting, that of his own internalized analysis, EU. Soumaki and D.C. Anagnostopoulos [27] argue that the statement often repeated that the analyst is the guarantor of the framework does not imply rigidity or coldness, given that analysts present their own psychic framework as the cornerstone of the analytic process, guaranteeing that they will assume full responsibility for it. On this, Abdel Malak [28], revisiting Winnicott, emphasizes the role of the psychotherapist and psychoanalyst to carry some of the functions of the frame themselves, notably those of a sense of continuity. 'Through my proposed options, I tried to preserve a continuity in discontinuity and testify to a presence in absence, despite the absence in presence, which would allow patients to 'experience separation without separation' [29]. The person of the therapist is responsible for taking over the functions of the frame when the meta-frame breaks in, fails, in times of exile, war or crisis. Khair Badawi [30] formulates that when the setting is inaccessible, its materiality is transposed onto the person of the analyst “who is no longer the guardian of the objective setting, but actually becomes that setting… Wherever the analyst happens to be. It is the articulation of the transference-countertransference relationship that structures the situation”.

Our participants admitted to feeling overwhelmed by the intrusion of external reality into their setting and mentioned losing their ability to contain and listen. Returning to analysis or supervision, as well as establishing a support system with peers, were ways for some of them to regain their ability to work with their patients. These findings are a response to questions from Bekes et al.'s [17] research on whether peer support and supervision during and after the pandemic could increase psychotherapists' ability to practice reflection as well as the resulting changes in their therapeutic work.

The extraordinary and traumatic circumstances affecting the Lebanese population are faced by psychotherapists and their patients. The impact of this shared trauma creates symmetry in the therapeutic relationship which is meant to be asymmetrical. Our findings concur with Nuttman-shwartz et al. [9] who state that shared traumatic situations can blur distinctions between therapists’ roles (personal/family vs. therapeutic) and can affect therapists' professional identity and professional effectiveness. In this, they are referring to Baum [31] whose research findings show that professionals working in shared traumatic realities suffer from lapses of empathy and professional distress with a direct impact on their personal growth.

Saidipour [32] introduces the term of “good enough therapy”, a metaphoric corollary to Winnicott’s concept of good enough mothering, when she tries to answer a question, she heard in professional venues but also asked herself: *How do we help our patients when we are living through a crisis with them?* A good enough therapist provides a holding environment and doesn’t thrive for perfection but only needs to be good enough and sensitive to their patient’s needs. She notes the increase in flexibility of the therapeutic setting in order to make room for one's own vulnerability. In this respect, Khoury Naja [33] insists on the therapist's duty to rethink the setting and to listen to his or her own intuition and creativity in order to offer patients a tailor-made support allowing the continuity of the therapeutic process.

Our results are consistent with the idea of creativity emerging from trauma. Our therapists' main purpose was to resume working with their patients. They adjusted their setting, reinterrogated their framework, used analysis, supervision and peer support. They were flexible and accepted new patients by working online to deal with the economic crisis.

### Strength and limitations of this study

#### Strengths

This study effectively analyzes the specificities of the Lebanese context, such as the economic crisis, political and security instability, and historical conflicts. By considering these factors, the article highlights the unique challenges faced by psychotherapists in Lebanon and the potential impact on the therapeutic relationship.

The study references previous research studies and literature reviews that have examined remote psychotherapy, therapeutic alliance, and online therapy. This demonstrates a solid understanding of existing knowledge on the subject and helps position the current study within the broader research landscape.

The study utilizes a qualitative research design, allowing for an in-depth exploration of the experiences and perspectives of therapists working with Lebanese patients during a socio-political and sanitary crisis. The data collected of the subjective experiences of psychotherapists answers partly the lack of qualitative research on the topic and helps enhance a deepened understanding of the therapists’ reactivity to crisis.

The inclusion criteria ensure that the participants have relevant professional experience and are directly impacted by the research topic. This sample size is reasonable for a qualitative study and allows for in-depth analysis. Additionally, the inclusion of participants with different therapy approaches adds diversity to the sample, enabling a broader understanding of the research topic.

### Limitations

The article focuses on the Lebanese context and does not extensively explore the practices of psychotherapists in other countries. However, it acknowledges the potential for comparative analysis, which will be the perspective of the analysis of the data collected in other contexts such as Brazil, Italy and France that had suffered the pandemic crisis along with socio-economic and political crisis.

This article does not explicitly address the potential impact of the researchers' own backgrounds, biases, or assumptions on the interpretation of the data. In order to answer this limitation, the researchers have resorted to inter-jury validation of the results at every step of the data analysis.

Overall, while the study offers valuable insights into the experiences of therapists working with Lebanese patients during a crisis, it is important to consider these strengths and limitations when interpreting and applying the findings.

## Conclusions

The aim of this study was to explore psychotherapists' lived experiences of working through multiple crises. The qualitative analysis of the therapists' interviews revealed a core aspect of their experiences: the feeling of insecurity resulting from the intrusion of external reality into their therapeutic setting and framework. The switch to online therapy, although a forced transition and for most participants a first experience, allowed them to continue working with their patients and even take on new patients. However, our results highlight the participants' uncertainty about the online setting and its impact on the therapeutic relationship.

Conducting online sessions from home led to a blending of personal and professional life, limiting reflection and tuning into sessions. Therapists made slight modifications to their methods and became more supportive, cautious, and ready to give advice while reducing fees. The study highlighted challenges to the therapy framework regarding frequency, fees, content, and resistance. Therapists relied on internalized analysis, assuming responsibility for the framework, and recognized the need to navigate the intrusion of external reality to explore its impact on patients' responses. Seeking support through analysis, supervision, and establishing a support system with peers was beneficial. "Good enough therapy" emerged as a concept, emphasizing the provision of a flexible, sensitive, and tailored holding environment to ensure continuity of the therapeutic process. The study emphasizes the need for adaptability, containment, and support in the experiences of psychotherapists working through multiple crises, particularly in the context of the online setting.

## Data Availability

The data are not publicly available due to privacy restrictions but are available from the corresponding author on reasonable request.

## Abbreviations

IPA: Interpretative Phenomenological Analysis
CBT: Cognitive behavioral therapy
EMDR: Eye movement desensibilisation and reprocessing
NLP: Neuro-linguistics programming
